# Subgroup analyses from the phase 3 ASCENT study of sacituzumab govitecan in metastatic triple-negative breast cancer

**DOI:** 10.1038/s41523-024-00635-5

**Published:** 2024-04-25

**Authors:** Sara A. Hurvitz, Aditya Bardia, Kevin Punie, Kevin Kalinsky, Lisa A. Carey, Hope S. Rugo, Véronique Diéras, See Phan, Rosemary Delaney, Yanni Zhu, Sara M. Tolaney

**Affiliations:** 1 Clinical Research Division, Department of Medicine, UW Medicine, Fred Hutchinson Cancer Center, Seattle, WA USA; 2 Massachusetts General Hospital Cancer Center, Harvard Medical School, Boston, MA USA; 3 Department of General Medical Oncology and Multidisciplinary Breast Centre, Leuven Cancer Institute and University Hospitals Leuven, Leuven, Belgium; 4 Winship Cancer Institute, Emory University, Atlanta, GA USA; 5 University of North Carolina Lineberger Comprehensive Cancer Center, Chapel Hill, NC USA; 6 University of California San Francisco, Helen Diller Family Comprehensive Cancer Center, San Francisco, CA USA; 7 Centre Eugène Marquis, Rennes, France; 8 Gilead Sciences Inc., Foster City, CA USA; 9 Dana-Farber Cancer Institute, Harvard Medical School, Boston, MA USA

**Keywords:** Breast cancer, Breast cancer

## Abstract

In this post hoc analysis of the ASCENT study, we compared outcomes with sacituzumab govitecan (SG) vs single-agent chemotherapy in clinically important subgroups of patients with metastatic triple-negative breast cancer (mTNBC). Patients with mTNBC refractory to/relapsing after ≥2 prior chemotherapies (≥1 in the metastatic setting) were randomized 1:1 to receive SG or treatment of physician’s choice (TPC) until unacceptable toxicity/progression. The primary endpoint was progression-free survival (PFS) per RECIST 1.1 by central review in patients without brain metastases. Patients with brain metastases were allowed if metastases were stable ≥4 weeks. In the intention-to-treat (ITT) population, 19% of patients were age ≥65 years; 12% were Black, and 12% had brain metastases. SG improved PFS and overall survival (OS), respectively, vs TPC in patients age ≥65 years (7.1 vs 2.4 months and 14.7 vs 8.9 months), or of Black race (5.4 vs 2.2 months and 13.8 vs 8.5 months), consistent with outcomes in the ITT population. Patients with brain metastases had numerically higher median PFS with SG vs TPC, but median OS was similar between treatment groups. SG was well tolerated and had a manageable safety profile consistent with the full safety population across all subgroups; neutropenia and diarrhea were the most common treatment-emergent adverse events. These findings confirm the meaningful clinical benefit of SG vs standard chemotherapy in patient subgroups with high unmet needs. SG should be considered an effective and safe treatment option for patients with mTNBC eligible for second-line or later therapy. ClinicalTrials.gov Number: NCT02574455.

## Introduction

Triple-negative breast cancer (TNBC) is a heterogenous disease with an aggressive clinical course and poorer outcomes than other breast cancer subtypes^1,2^. Certain patient subgroups defined by age, race, or location of disease (e.g., brain metastases) within the TNBC subtype are associated with even worse outcomes. Approximately 20% of new TNBC diagnoses are in patients age ≥65 years, who may have a higher rate of comorbidities, complicating their ability to tolerate systemic treatment-related toxicities^3–6^. Incidence rates of metastatic TNBC (mTNBC) among Black women are double those of White women, with worse clinical outcomes, potentially due to healthcare disparities, comorbidities, and differences in disease biology^7–9^. Patients with mTNBC who have brain metastases can have debilitating neurologic symptoms and poor survival, and treatment options may be limited due to challenges with drug delivery across the blood–brain barrier^10^.

Though the treatment landscape for mTNBC has evolved, patients with later-line mTNBC, especially in these poor prognosis subgroups, exclusively relied on single-agent chemotherapy as the standard treatment option until recently. However, single-agent chemotherapy is associated with low response rates, short progression‑free survival (PFS), and dose-limiting, cumulative adverse events (AEs)^11–16^. As a result, these patients could benefit from more effective and well-tolerated novel agents.

Sacituzumab govitecan (SG) is an antibody-drug conjugate (ADC) composed of anti–trophoblast cell surface antigen 2 (Trop-2) antibody coupled to the well-characterized payload SN-38 via a proprietary, hydrolyzable linker. Trop-2 is highly expressed in all breast cancer subtypes and has been shown to be a viable target in TNBC^17,18^. SG is approved in multiple countries (including the United States) for patients with mTNBC who received ≥2 prior therapies (≥1 in the metastatic setting) and is also approved in the United States for patients with hormone receptor positive/human epidermal growth factor receptor (HER2) negative metastatic breast cancer who received endocrine-based therapy and ≥2 additional systemic therapies in the metastatic setting^19–21^. Approval for mTNBC was based on results from the global, open-label, phase 3 ASCENT study (NCT02574455), which demonstrated a significant survival improvement for SG vs single-agent chemotherapy, with a manageable safety profile in the second-line or later mTNBC setting^22,23^. Median PFS was 4.8 vs 1.7 months (hazard ratio [HR] 0.43; 95% confidence interval [CI] 0.35–0.54), and median overall survival (OS) was 11.8 vs 6.9 months (HR 0.51; 95% CI 0.41–0.62) in the intention-to-treat (ITT) population^22^.

Given the proven clinical benefit of SG vs single-agent chemotherapy in the mTNBC second-line or later setting, it is important to further understand whether specific patient subgroups could derive benefit from SG. Here, we present post hoc efficacy and safety results from the ASCENT study in patients with mTNBC by subgroups based on age, race, presence of previously treated brain metastases, and by chemotherapy treatment of physician’s choice (TPC) selected prior to randomization in the ITT population.

## Results

### Patients

In total, 529 patients enrolled in the study (ITT population) were randomly assigned to SG (*n* = 235) or TPC (*n* = 233; 54% eribulin, 20% vinorelbine, 13% capecitabine, or 12% gemcitabine); 468 patients had no known brain metastases at baseline^22^.

Baseline patient demographics and disease characteristics for the ITT population and by subgroup are shown in Table 1. In the ITT population, the median age was 54 years, and the median number of prior lines of systemic therapy was four. Of the 529 patients, 101 patients (19%) were age ≥65 years, 62 patients (12%) self-reported Black race, and 61 patients (12%) had known brain metastases at baseline.

**Table 1:** Baseline patient and disease characteristics and prior treatment history for the ITT population and patient subgroups of age, race, or brain metastases

Characteristic	ITT (*N* = 529)	Age		Race		Brain metastases	
		<65 years (*n* = 428)	≥65 years (*n* = 101)	Black (*n* = 62)	Other race (*n* = 467)	No (*n* = 468)	Yes (*n* = 61)
Female, *n* (%)	527 (<99)	427 (>99)	100 (99)	62 (100)	465 (99)	466 (99)	61 (100)
Median age, years (range)	54 (27–82)	51 (27–64)	70 (65–82)	54 (32–75)	54 (27–82)	54 (27–82)	53 (27–81)
Race or ethnic group, *n* (%)^a^							
White	418 (79)	332 (78)	86 (85)	0	418 (79)	369 (79)	49 (80)
Black	62 (12)	53 (12)	0	62 (100)	0	56 (12)	6 (10)
Asian	22 (4)	22 (5)	0	0	22 (4)	18 (4)	4 (7)
Other or not specified	27 (5)	21 (5)	6 (6)	0	27 (5)	25 (5)	2 (3)
ECOG performance status at screening, *n* (%)^b^							
0	229 (43)	187 (44)	42 (42)	26 (42)	203 (44)	206 (44)	23 (38)
1	300 (57)	241 (56)	59 (58)	36 (58)	264 (57)	262 (56)	38 (62)
Known brain metastases at entry study, *n* (%)	61 (12)	50 (12)	11 (11)	6 (10)	55 (10)	0	61 (100)
Germline *BRCA*1/2 mutational status, *n* (%)^c^							
Negative	296 (56)	261 (61)	35 (35)	30 (48)	266 (57)	258 (55)	38 (62)
Positive	43 (8)	37 (9)	6 (6)	2 (3)	41 (9)	34 (7)	9 (15)
Missing	190 (36)	130 (30)	60 (59)	30 (48)	160 (35)	176 (38)	14 (23)
Triple-negative breast cancer at initial diagnosis, *n* (%)^d^	372 (70)	305 (71)	67 (66)	49 (79)	323 (69)	322 (69)	50 (82)
Median time from metastatic diagnosis, months (range)^e^	16 (0–203)	16 (0–140)	19 (0–203)	20 (1–96)	16 (0–203)	15 (0–203)	23 (2–96)
Number of prior anticancer regimens, median (range)^f^	3 (1–16)	3 (1–16)	3 (1–10)	3 (1–9)	3 (1–16)	3 (1–16)	4 (2–9)
Number of prior chemotherapies, *n* (%)							
2–3	365 (69)	300 (70)	65 (64)	43 (69)	322 (69)	330 (71)	35 (57)
>3	164 (31)	128 (30)	36 (36)	19 (31)	145 (31)	138 (30)	26 (43)
Most common prior chemotherapy drugs, *n* (%)							
Taxanes^g^	529 (100)	428 (100)	101 (100)	62 (100)	467 (100)	468 (100)	61 (100)
Anthracyclines^h^	438 (83)	370 (86)	68 (67)	51 (82)	387 (83)	384 (82)	54 (89)
Cyclophosphamide	437 (83)	363 (85)	74 (73)	50 (81)	387 (83)	384 (82)	53 (87)
Carboplatin	343 (65)	284 (66)	59 (58)	48 (77)	311 (67)	307 (66)	36 (59)
Capecitabine	354 (67)	284 (66)	70 (69)	43 (69)	295 (63)	306 (65)	48 (79)
Previous use of checkpoint inhibitors, *n* (%)	153 (29)	130 (30)	23 (23)	23 (37)	130 (28)	127 (27)	26 (43)
Previous use of PARP inhibitors, *n* (%)	42 (8)	37 (9)	5 (5)	3 (5)	39 (8)	35 (8)	7 (11)
Number of prior systemic regimens, median (range)	4 (2–17)	4 (2–17)	4 (2–11)	4 (2–10)	4 (2–17)	4 (2–17)	5 (2–10)
Setting of prior systemic therapies (%)							
Adjuvant	309 (58)	244 (57)	65 (64)	33 (53)	276 (52)	269 (58)	40 (66)
Neoadjuvant	249 (47)	225 (53)	24 (24)	29 (47)	220 (47)	224 (48)	25 (41)
Metastatic	518 (98)	417 (97)	101 (100)	59 (95)	459 (98)	457 (98)	61 (100)
Locally advanced disease	15 (3)	14 (3)	1 (1)	2 (3)	13 (3)	12 (3)	3 (5)
Major tumor locations based on IRC, *n* (%)^i^							
Lung	246 (47)	194 (45)	52 (52)	29 (47)	217 (47)	205 (44)	41 (67)
Bone^j^	125 (24)	101 (24)	24 (24)	14 (23)	111 (24)	103 (22)	22 (36)
Liver	221 (42)	183 (43)	38 (38)	24 (39)	197 (42)	199 (43)	22 (36)
Axillary lymph nodes	137 (26)	114 (27)	23 (23)	23 (37)	114 (24)	130 (28)	7 (11)

ITT population comprises patients with and without brain metastases at baseline. The Black, Other race, and age subgroup analyses were conducted in the ITT population.

*BRCA* breast cancer gene, *ECOG* Eastern Cooperative Oncology Group, *HER2* human epidermal growth factor receptor 2, *HR* hormone receptor, *IRC* independent review committee, *ITT* intention to treat, *PARP* poly (adenosine diphosphate-ribose) polymerase, *SG* sacituzumab govitecan, *TPC* treatment of physician’s choice.

^a^Race was self-reported. Other includes American Indian or Alaska Native, Native Hawaiian or other Pacific Islander. Other race subgroup includes any patient who did not self-identify as Black race.

^b^The Eastern Cooperative Oncology Group performance status scale ranges from 0 to 5: 0 indicates that the patient was fully active with no restrictions and 1 indicates that the patient was ambulatory and able to carry out work of a light or sedentary nature but restricted in physically strenuous activity. Higher numbers indicate increasing degrees of disability.

^c^Patients who did not have *BRCA*1/2 germline testing done or had inconclusive results are not included. Of the patients with *BRCA*1/2 mutations at baseline, 10 (63%) in the SG group and 11 (61%) in the TPC group had received prior PARP inhibitors.

^d^Patients initially diagnosed with HR-positive and/or HER2-positive breast cancer (SG, *n* = 70; TPC, *n* = 76) had a median (range) time from metastatic diagnosis of 22.5 months (2.1–202.9) in the SG group and 21.2 months (1.1–140.1) in the TPC group.

^e^Time from metastatic diagnosis is defined as number of days divided by 30.4375 from date of first diagnosis of metastasis to date of study entry.

^f^Anticancer regimens refer to any prior metastatic/neoadjuvant/locally advanced regimens used to treat an eligible breast cancer patient. Prior therapy in the adjuvant setting is excluded from this count.

^g^Includes paclitaxel, nab-paclitaxel, and docetaxel.

^h^Includes doxorubicin, daunorubicin, epirubicin, and different formulations of these agents.

^i^Based on independent central review of target and nontarget lesions at baseline.

^j^Bone-only disease was not permitted in the study.

In general, patient disease characteristics were similar between patients aged <65 years (*n* = 428) and ≥65 years (*n* = 101) with some exceptions. Patients aged <65 years had a higher rate (61%) of negative germline breast cancer gene (*BRCA*) mutations in those patients with known *BRCA* status than patients aged ≥65 years (35%). The most common prior chemotherapy treatment regimens were generally used at a higher frequency with patients aged <65 vs ≥65 years, including anthracyclines (86% vs 67%, respectively) and cyclophosphamide (85% vs 73%); differences were also found in the use of previous checkpoint inhibitors (30% vs 23%) and neoadjuvant systemic therapies (53% vs 24%) (Table 1).

Baseline characteristics between Black (*n* = 62) and Other race (*n* = 467) subgroups were similar except for missing information regarding *BRCA*1/2 mutation status (48% vs 35%, respectively), a higher rate of TNBC at initial diagnosis (79% vs 69%), and a higher frequency of axillary lymph node involvement (37% vs 24%). Additionally, there were some differences in the most common prior chemotherapy treatments between Black and Other subgroups, in particular for carboplatin (77% vs 67%). Black patients also had higher previous use of checkpoint inhibitors (37%) vs Other race patients (28%) (Table 1).

In general, patient disease characteristics were similar between patients without (*n* = 468) and with (*n* = 61) brain metastases with some exceptions. Patients with brain metastases had a higher rate of *BRCA* mutations (15%) than patients without brain metastases (7%). More patients with vs without brain metastases had TNBC at initial diagnosis (82% vs 69%, respectively), a greater number of prior anticancer regimens (median [range]: 4 [2–9]) vs 3 [1–16]), a higher rate of >3 prior chemotherapies (43% vs 30%), prior capecitabine therapy (79% vs 65%), and previous checkpoint inhibitory treatment (43% vs 27%) prior to enrollment. Patients with brain metastases also more commonly had major tumor locations in the lung (67% vs 44%) and bone (36% vs 22%), and less commonly in the liver (36% vs 43%) and axillary lymph nodes (11% vs 28%) (Table 1).

Demographics and baseline characteristics for SG and each TPC agent were balanced between treatment arms. Within the TPC arm, the most commonly used chemotherapy was eribulin (*n* = 139), followed by vinorelbine (*n* = 52), capecitabine (*n* = 33), and gemcitabine (*n* = 38) (Table 2).

**Table 2:** Baseline patient and disease characteristics in SG or individual TPC agent selected at randomization

Characteristic	Treatment				
	SG (*n* = 267)	Eribulin (*n* = 139)	Vinorelbine (*n* = 52)	Capecitabine (*n* = 33)	Gemcitabine (*n* = 38)
Female, *n* (%)	265 (99)	139 (100)	52 (100)	33 (100)	38 (100)
Median age, years (range)	54 (27–82)	53 (27–80)	54 (30–74)	50 (31–81)	56 (36–81)
Race or ethnic group, *n* (%)^a^					
White	215 (81)	109 (78)	36 (69)	27 (82)	31 (82)
Black	28 (11)	18 (13)	12 (23)	2 (6)	2 (5)
Asian	13 (5)	3 (2)	2 (4)	3 (9)	1 (3)
Other or not specified	11 (4)	9 (7)	2 (4)	1 (3)	4 (11)
ECOG performance status at screening, *n* (%)^b^					
0	121 (45)	63 (45)	22 (42)	13 (39)	10 (26)
1	146 (55)	76 (55)	30 (58)	20 (61)	28 (74)
Number of prior chemotherapies (*n*, %)					
2–3	99	57	4	18	13
>3	168	82	48	15	25
Number of prior systemic regimens, median (range)	4 (2–17)	4 (2–14)	5 (2–14)	3 (2–7)	5 (2–10)

ITT population comprises patients with and without brain metastases at baseline. The Black, Other Race, and age subgroup analyses were conducted in the ITT population.

*BRCA* breast cancer gene, *ECOG* Eastern Cooperative Oncology Group, *ITT* intention to treat, *SG* sacituzumab govitecan, *TPC* treatment of physician’s choice.

^a^Race was self-reported. Other includes American Indian or Alaska Native, Native Hawaiian or other Pacific Islander. Other Race subgroup includes any patient who did not self-identify as Black race.

^b^The Eastern Cooperative Oncology Group performance status scale ranges from 0 to 5: 0 indicates that the patient was fully active with no restrictions and 1 indicates that the patient was ambulatory and able to carry out work of a light or sedentary nature but restricted in physically strenuous activity. Higher numbers indicate increasing degrees of disability.

**Fig. 1:**
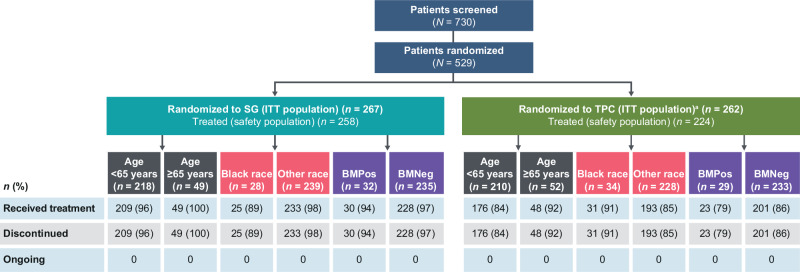
CONSORT diagram. ^a^Patients in the TPC arms were randomized to eribulin (*n* = 139), vinorelbine (*n* = 52), gemcitabine (*n* = 38), or capecitabine (*n* = 33). Details of the trial profile for the BMNeg population have been published previously. Reasons the patients discontinued treatment in each subgroup are presented in Table S1 in the Supplemental Appendix. Other race subgroup includes any patient who did not self-identify as Black race. BMNeg brain metastases-negative, BMPos brain metastases-positive, ITT intention to treat, SG sacituzumab govitecan, TPC treatment of physician’s choice.

In total, 258 patients in the SG group and 224 patients in the TPC group were treated. As of February 25, 2021, no patients remained on treatment in any subgroup. Across subgroups, disease progression was the most common reason for treatment discontinuation for SG and TPC (age ≥65 years, 88% and 75%; Black race, 79% and 74%; with brain metastases, 72% and 62% for SG and TPC, respectively). The median duration of follow-up for all ITT patients was 8.8 months. Median duration of SG treatment varied across the subgroups (Supplementary Table 1). Patients age ≥65 years generally had the longest exposure to SG treatment (median, 6.7 months) and patients with brain metastases had the shortest (median, 2.5 months). Patient disposition for the patient subgroups is presented in Fig. 1 and Supplementary Table 1.

### Efficacy outcomes

#### Age

**Fig. 2:**
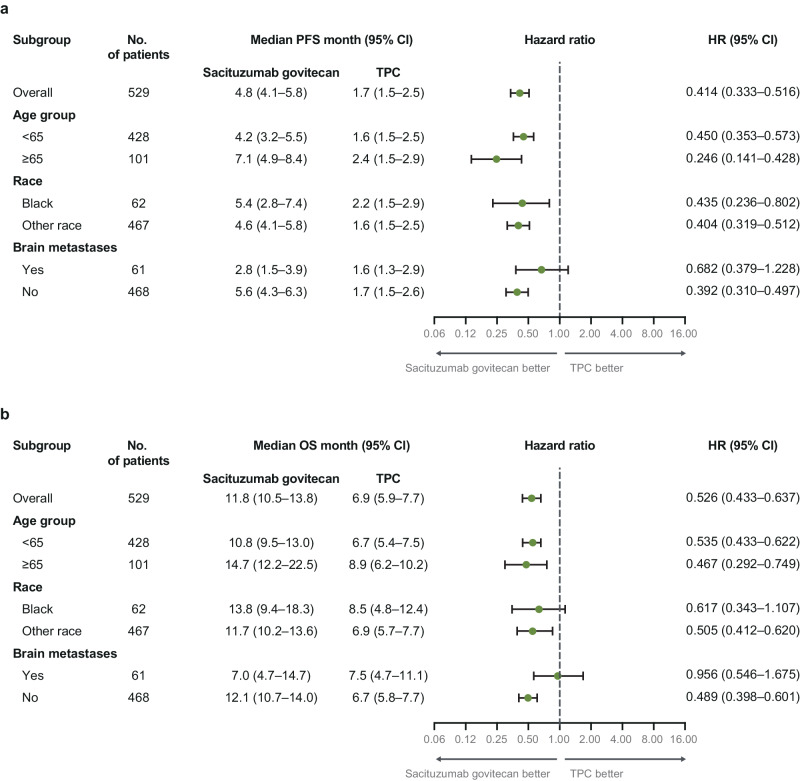
Subgroup analysis of progression-free survival and overall survival. **a** Progression-free survival; **b** overall survival. Survival outcomes were assessed in the intention-to-treat population (all randomly assigned patients with and without brain metastases). Other race subgroup includes any patient who did not self-identify as Black race. PFS was determined by blinded independent central review according to Response Evaluation Criteria in Solid Tumors, version 1.1. OS overall survival, PFS progression-free survival, SG sacituzumab govitecan, TPC treatment of physician’s choice.

For patients <65 years (*n* = 428), median PFS for SG vs TPC was 4.2 vs 1.6 months (HR 0.45; 95% CI 0.35–0.57), and median OS was 10.8 vs 6.7 months (HR 0.54; 95% CI 0.43–0.66), respectively (Fig. 2). Objective response rate (ORR) was 28% vs 5%, and clinical benefit rate (CBR) was 37% vs 8%, respectively, in these patients (Table 3). In patients age ≥65 years (*n* = 101), median PFS for SG vs TPC was 7.1 vs 2.4 months (HR 0.25; 95% CI 0.14–0.43), and median OS was 14.7 vs 8.9 months (HR 0.47; 95% CI 0.29–0.75; Table  2 and Fig. 2). ORR by blinded independent central review (BICR) was 45% vs 0%, and CBR was 55% vs 8%, respectively (Table 3).

**Table 3:** Summary of efficacy outcomes for age, race, or brain metastases

	Age				Race				Brain metastases			
	<65 years (*n* = 428)		≥65 years (*n* = 101)		Black (*n* = 62)		Other race (*n* = 467)		No (*n* = 468)		Yes (*n* = 61)	
	SG (*n* = 218)	TPC (*n* = 210)	SG (*n* = 49)	TPC (*n* = 52)	SG (*n* = 28)	TPC (*n* = 34)	SG (*n* = 239)	TPC (*n* = 228)	SG (*n* = 235)	TPC (*n* = 233)	SG (*n* = 32)	TPC (*n* = 29)
Median PFS, mo (95% CI)	4.2 (3.2–5.5)	1.6 (1.5–2.5)	7.1 (4.9–8.4)	2.4 (1.5–2.9)	5.4 (2.8–7.4)	2.2 (1.5–2.9)	4.6 (4.1–5.8)	1.6 (1.5–2.5)	5.6 (4.3–6.3)	1.7 (1.5–2.5)	2.8 (1.5–3.9)	1.6 (1.3–2.9)
HR (95% CI)	0.45 (0.35–0.57)		0.25 (0.14–0.43)		0.44 (0.24–0.80)		0.40 (0.32–0.51)		0.45 (0.31–0.494)		0.68 (0.38–1.23)	
Median OS, mo (95% CI)	10.8 (9.5–13.0)	6.7 (5.4–7.5)	14.7 (12.2–22.5)	8.9 (6.2–10.2)	13.8 (9.4–18.3)	8.5 (4.8–12.4)	11.7 (10.2–13.6)	6.9 (5.7–7.7)	12.1 (10.7–14.0)	6.7 (5.8–7.7)	7.0 (4.7–14.7)	7.5 (4.7–11.1)
HR (95% CI)	0.54 (0.43–0.66)		0.47 (0.29–0.75)		0.62 (0.34–1.11)		0.51 (0.41–0.62)		0.49 (0.4–0.60)		0.96 (0.55–1.68)	
ORR, n (%)	61 (28)	11 (5)	22 (45)	0	9 (32)	2 (6)	74 (31)	9 (4)	82 (35)	11 (5)	1 (3)	0
Best overall response n (%)												
CR	7 (3)	2 (1)	3 (6)	0	1 (4)	1 (3)	9 (4)	1 (0.4)	10 (4)	2 (1)	0	0
PR	54 (25)	9 (4)	19 (39)	0	8 (29)	1 (3)	65 (27)	8 (4)	72 (31)	9 (4)	1 (3)	0
SD	76 (35)	49 (23)	20 (41)	22 (42)	11 (39)	12 (35)	85 (36)	59 (26)	81 (34)	62 (27)	15 (47)	9 (31)
SD ≥ 6 mo	20 (9)	6 (3)	5 (10)	4 (8)	3 (11)	3 (9)	22 (9)	7 (3)	23 (10)	9 (4)	2 (6)	1 (3)
PD	58 (27)	82 (39)	7 (14)	18 (35)	4 (14)	14 (41)	61 (26)	86 (38)	54 (23)	89 (38)	11 (34)	11 (38)
NE^a^	23 (11)	68 (32)	0	12 (23)	4 (14)	6 (18)	19 (8)	74 (33)	18 (8)	71 (30)	5 (16)	9 (31)
CBR, n (%)^b^	81 (37)	17 (8)	27 (55)	4 (8)	12 (43)	5 (15)	96 (40)	16 (7)	105 (45)	20 (9)	3 (9)	1 (3)
Median DOR, mo (95% CI)^c^	5.6 (5.1–7.6)	3.6 (2.8–NE)	7.1 (4.4–12.3)	N/A	9.2 (3.2–NE)	NE (2.9–NE)	5.7 (5.4, 7.9)	3.6 (2.8, NE)	6.3 (5.5–7.9)	3.6 (2.8–NE)	2.9 (NE–NE)	N/A^d^
Median TTR, mo (range)	1.5 (0.7–10.6)	1.5 (1.3–4.2)	1.5 (1.2–8.4)	N/A	1.4 (1.3–10.6)	2.2 (1.4–3.0)	1.6 (0.7–8.4)	1.5 (1.3–4.2)	1.5 (0.7–10.6)	1.5 (1.3–4.2)	1.5 (1.5–1.5)	0

Other race subgroup includes any patient who did not self-identify as Black race.

*CBR* clinical benefit rate, *CI* confidence interval, *CR* complete response, *DOR* duration of response, *HR* hazard ratio, *NE* not estimable, *ORR* objective response rate, *OS* overall survival, *PD* progressive disease, *PFS* progression-free survival, *PR* partial response, *SD* stable disease, *SG* sacituzumab govitecan, *TPC* treatment of physician’s choice, *TTR* time to response.

^a^Response could not be evaluated for a variety of reasons, including a lack of postbaseline images or unreadable images.

^b^CBR is defined as the percentage of patients with a confirmed overall response of CR or PR and SD ≥ 6 months.

^c^Median duration of response is from Kaplan–Meier estimate. CI for median is computed using the Brookmeyer–Crowley method.

^d^No patients to report.

#### Black race

In Black patients (SG, *n* = 28; TPC, *n* = 34), median PFS for SG vs TPC was 5.4 vs 2.2 months (HR 0.44; 95% CI 0.24–0.80), and median OS was 13.8 vs 8.5 months (HR 0.62; 95% CI 0.34–1.11). ORR by BICR was 32% vs 6%, and CBR was 43% and 15%, respectively. In the SG group, one patient (4%) achieved a complete response (CR), and eight patients (29%) achieved a partial response (PR). In Other race patients (SG, *n* = 239; TPC *n* = 228), median PFS for SG vs TPC was 4.6 vs 1.6 months (HR 0.40; 95% CI 0.32–0.51), and median OS was 11.7 vs 6.9 months (HR 0.51; 95% CI 0.41–0.62). ORR by BICR was 31% vs 4%, and CBR was 40% vs 7%, respectively. In the SG group, 9 patients (4%) achieved a CR, and 65 patients (27%) achieved a PR (Table 3).

#### Brain metastases

In patients with stable brain metastases at study entry (SG, *n* = 32; TPC, *n* = 29), median PFS for SG vs TPC was 2.8 vs 1.6 months (HR 0.68; 95% CI 0.38–1.23). Median OS was 7.0 vs 7.5 months (HR 0.96; 95% CI 0.55–1.68; Table 3). ORR by BICR was 3% vs 0%, and CBR was 9% and 3%, respectively (Table 3).

In patients without brain metastases at study entry (SG, *n* = 235; TPC, *n* = 233), median PFS for SG vs TPC was 5.5 vs 1.7 months (HR 0.35; 95% CI 0.28–0.44). Median OS was 12.1 vs 6.7 months (HR 0.48; 95% CI 0.38–0.59). ORR by BICR was 35% vs 5%, and CBR was 45% vs 9%, respectively (Table 3).

#### Individual TPC agents

Treatment with SG (*n* = 267) resulted in longer median PFS vs eribulin (*n* = 139), vinorelbine (*n* = 52), capecitabine (*n* = 33), or gemcitabine (*n* = 38), with a median of 4.8 vs 2.1, 1.5, 1.6, and 2.4 months, respectively, as well as OS (median 11.8 vs 7.2, 5.6, 5.2, and 8.4 months) and ORR (31% vs 4%, 4%, 6%, and 3%, respectively) (Table 4).

**Table 4:** Summary of efficacy outcomes with SG or individual TPC agent selected at randomization

Treatment						
		TPC				
	SG (*n* = 267)	Eribulin (*n* = 139)	Vinorelbine (*n* = 52)	Capecitabine (*n* = 33)		Gemcitabine (*n* = 38)
Median PFS, mo (95% CI)	4.8 (4.1–5.8)	2.1 (1.5–2.8)	1.5 (1.4–2.5)	1.6 (1.4–2.4)		2.4 (1.4–2.9)
HR (95% CI)^a^			0.41 (0.33–0.52)			
Median OS, mo (95% CI)	11.8 (10.5–13.8)	7.2 (6.2–8.2)	5.6 (3.5–6.7)	5.2 (3.5–8.6)		8.4 (5.0–9.4)
HR (95% CI)^a^			0.51 (0.42–0.62)			
ORR, n (%)	83 (31)	6 (4)	2 (4)	2 (6)		1 (3)
Best overall response, n (%)						
CR	10 (4)	2 (1)	0	0		0
PR	73 (27)	4 (3)	2 (4)	2 (6)		1 (3)
SD	96 (36)	39 (28)	10 (19)	8 (24)		14 (37)
SD ≥ 6 mo	25 (9)	4 (3)	2 (4)	1 (3)		3 (8)
PD	65 (24)	57 (41)	21 (40)	13 (39)		9 (24)
NE^b^	23 (9)	37 (27)	19 (37)	10 (30)		14 (37)
CBR, n (%)^c^	108 (40)	10 (7)	4 (8)	3 (9)		4 (11)
Median DOR, mo (95% CI)^d^	6 (6–8)	4 (3–NE)	3 (NE)	NA		3 (NE)
Median TTR, mo (range)	2 (1–11)	1 (1–3)	1 (1–2)	3 (1–4)		2 (2–2)

SG data have been previously published: Bardia et al.^22^.

*CBR* clinical benefit rate, *CI* confidence interval, *CR* complete response, *DOR* duration of response, *HR* hazard ratio, *NE* not estimable, *ORR* objective response rate, *OS* overall survival, *PD* progressive disease, *PFS* progression-free survival, *PR* partial response, *SD* stable disease, *SG* sacituzumab govitecan, *TPC* treatment of physician’s choice, *TTR* time to response.

^a^Hazard ratio analysis based on comparison of SG vs total TPC arm. Stratified Cox regression adjusted for stratification factors: number of prior chemotherapies, presence of known brain metastases at study entry, and region.

^b^Response could not be evaluated for a variety of reasons, including a lack of postbaseline images or unreadable images.

^c^CBR is defined as the percentage of patients with a confirmed overall response of CR or PR and SD ≥ 6 months.

^d^Median duration of response is from Kaplan–Meier estimate. CI for median is computed using the Brookmeyer–Crowley method.

### Safety outcomes

In general, the safety profile of SG in the overall safety population was similar to that for TPC and was manageable. Grade ≥3 treatment-emergent AEs (TEAEs) occurred in 73% of patients treated with SG and 65% of patients treated with TPC. Serious AEs occurred in 27% and 29% of patients treated with SG compared with TPC, respectively. TEAEs leading to dose reduction occurred in 22% of patients treated with SG compared with 26% of patients treated with TPC, and 5% of patients in both arms experienced TEAEs leading to study drug discontinuation (Table 5).

**Table 5:** Safety summary for patient subgroups patient subgroups of age, race, or brain metastases

	Overall safety population (*n* = 482)		Age				Race				Brain metastases			
			<65 years (*n* = 385)		≥65 years (*n* = 97)		Black (*n* = 56)		Other race (*n* = 426)		No (*n* = 429)		Yes (*n* = 53)	
	SG (*n* = 258)	TPC (*n* = 224)	SG (*n* = 209)	TPC (*n* = 176)	SG (*n* = 49)	TPC (*n* = 48)	SG (*n* = 25)	TPC (*n* = 31)	SG (*n* = 233)	TPC (*n* = 193)	SG (*n* = 228)	TPC (*n* = 201)	SG (*n* = 30)	TPC (*n* = 23)
Any TEAE, *n* (%)	257 (100)	219 (98)	208 (100)	171 (97)	49 (100)	48 (100)	25 (100)	31 (100)	232 (99)	188 (97)	227 ( > 99)	196 (98)	30 (100)	23 (100)
Grade ≥3	188 (73)	145 (65)	154 (74)	115 (65)	34 (69)	30 (63)	18 (72)	22 (71)	170 (73)	123 (64)	164 (72)	129 (64)	24 (80)	16 (70)
Leading to dose reduction	57 (22)	59 (26)	39 (19)	43 (24)	18 (37)	16 (33)	7 (28)	11 (36)	50 (22)	48 (25)	52 (23)	51 (25)	5 (17)	8 (35)
Leading to Study drug interruption	162 (63)	87 (39)	137 (66)	66 (38)	25 (51)	21 (44)	16 (64)	15 (48)	146 (63)	72 (37)	143 (63)	78 (39)	19 (63)	9 (39)
Leading to treatment discontinuation	12 (5)	12 (5)	11 (5)	11 (6)	1 (2)	1 (2)	1 (4)	1 (3)	11 (5)	11 (6)	10 (4)	10 (5)	2 (7)	2 (9)
Leading to death	1 (0)	3 (1)	0	3 (2)	1 (2)	0	0	1 (3)	1 (0.4)	2 (1)	1 (<1)	2 (1)	0	1 (4)
Any SAE	69 (27)	64 (29)	57 (27)	47 (27)	12 (24)	17 (35)	5 (20)	8 (26)	64 (28)	56 (29)	60 (26)	54 (27)	9 (30)	10 (44)

Other race subgroup includes any patient who did not self-identify as Black race.

*SAE* serious adverse event, *SG* sacituzumab govitecan, *TPC* treatment of physician’s choice, *TEAE* treatment-emergent adverse event.

Overall, a similar AE profile was seen for SG among the age, race, and brain metastases subgroups. In the SG arm, the frequency of grade ≥3 TEAEs was higher in patients with brain metastases vs patients without brain metastases (80% vs 72%). Patients of Black race in the TPC arm were more likely to have an AE leading to dose reduction (35% vs 25%) or interruption (48% vs 37%) compared with Other race patients. Patients age ≥65 years were more likely to undergo dose reduction due to TEAEs vs patients age <65 years (37% vs 19%). The incidence of TEAEs leading to SG treatment discontinuation was generally low across subgroups (age ≥65 years, 2%; of Black race, 4%; with brain metastases, 7%) and consistent with that of the overall safety population (5%). Serious AEs occurred less frequently in patients of Black race compared with the overall safety population (20% vs 27%).

The most common TEAEs of any grade with SG vs TPC in the overall safety population included diarrhea (65% vs 17%), neutropenia (64% vs 44%), nausea (62% vs 30%), and fatigue (52% vs 40%). These were also the most common TEAEs of any grade across the patient subgroups, all of which occurred at a higher frequency in the SG vs TPC arm: neutropenia (age ≥65 years, 59% vs 44%; Black race, 64% vs 61%; with brain metastases, 63% vs 52%, respectively), diarrhea (age ≥65 years, 74% vs 19%; Black race, 72% vs 19%; with brain metastases, 50% vs 13% respectively), nausea (age ≥65 years, 51% vs 29%; Black race, 52% vs 35%; with brain metastases, 43% vs 26%, respectively), and fatigue (aged ≥65 years, 53% vs 50%; Black race, 52% vs 45%; with brain metastases, 63% vs 52%, respectively) (Supplementary Results and Supplementary Tables 2–4).

The most common grade ≥3 TEAEs in the SG arm vs TPC arm in the overall safety population were neutropenia (52% vs 34%) and diarrhea (12% vs <1%). The same grade ≥3 TEAEs were the most common across the patient subgroups, and occurred at a higher frequency in the SG vs TPC arm: neutropenia (age ≥65 years, 47% vs 40%; Black race, 48% vs 42%; with brain metastases, 60% vs 26%, respectively) and diarrhea (age ≥65 years, 12% vs 0%; Black race, 4% vs 0% with brain metastases, 7% vs 0%, respectively) (Supplementary Results and Supplementary Tables 2–4). In the SG arm, concomitant growth factor support and other supportive measures were used for AE management as previously described^22^. No interstitial lung disease was observed in any subgroup.

#### SG and TPC

Grade ≥3 TEAEs with SG vs eribulin or vs vinorelbine, capecitabine, and gemcitabine combined were primarily hematological. Discontinuation rates due to TEAE were generally similar between groups (Table 6 and Supplemental Table 5).

**Table 6:** Safety summary with SG or individual TPC agent selected at randomization

	Treatment				
	SG (*n* = 258)	Eribulin (*n* = 122)	Vinorelbine (*n* = 43)	Capecitabine (*n* = 28)	Gemcitabine (*n* = 31)
Any TEAE, *n* (%)	257 (99)	119 (98)	42 (98)	27 (96)	31 (100)
Grade ≥3					
Leading to dose reduction	57 (22)	27 (22)	17 (40)	3 (11)	12 (40)
Leading to dose interruption	162 (63)	37 (30)	27 (63)	7 (25)	16 (52)
Leading to treatment discontinuation	12 (5)	3 (3)	4 (10)	2 (7)	3 (10)
Leading to death	0	1 (1)	0	0	0
Any SAE	69 (27)	32 (26)	15 (35)	8 (27)	9 (29)

*SAE* serious adverse event, *SG* sacituzumab govitecan, *TPC* treatment of physician’s choice, *TEAE* treatment-emergent adverse event.

## Discussion

Due to the significant clinical benefit observed in the phase 3 ASCENT study, SG was approved for use and recommended by major guidelines for the treatment of patients with mTNBC who received ≥2 prior therapies (≥1 in the metastatic setting)^19–21,24^. Additional subgroup analyses for specific patient populations with pretreated mTNBC that have high unmet need and present particular challenges provide valuable insight into SG outcomes for treatment decision making. In these post hoc analyses of the ASCENT study, SG demonstrated improved outcomes compared with single-agent chemotherapy among patients who are age ≥65 years, of Black race, or with brain metastases, with a safety profile consistent with the original ITT population^22^. Furthermore, SG showed consistent efficacy benefit over each TPC chemotherapy agent individually including median PFS, OS, and ORR.

Comorbidities and functional impairment can predispose patients to a higher rate of chemotherapy-related toxicities, especially in frail older patients with TNBC^3,4,25^. In ASCENT, patients age ≥65 years who received SG had a significant improvement in outcomes compared to TPC with respect to PFS (median, 7.1 vs 2.4 months), OS (median, 15.3 vs 8.2 months), and ORR (50% vs 0%). This efficacy benefit together with longer time to treatment discontinuation for SG vs TPC in the ITT population^26^, supports a favorable risk/benefit profile for SG vs TPC in patients with mTNBC age ≥65 years.

Black patients with mTNBC have poor outcomes but historically constituted a low percentage of breast cancer clinical trial participants (3%–5%)^7–9^. As a result, data related to optimal treatment of these patients are often lacking, though efforts are ongoing to improve representation in clinical trials^27^. In total, 62 (12%) patients enrolled in the ASCENT study self-identified as Black. Black patients derived a similar clinical benefit from SG over TPC in PFS (Black: 5.4 vs 2.2 months; Other: 4.6 vs 1.6 months) and OS (Black: 13.8 vs 8.5 months; Other: 11.7 vs 6.9 months) as seen in the Other race population, suggesting SG is an effective treatment option for these patients.

Clinical trials in patients with breast cancer often exclude patients with brain metastases due to their poor prognosis and the limited ability of systemic agents to cross the blood–brain barrier; as such, limited clinical data are available for these patients^10^. Translational data suggest that SN-38 can cross the blood–brain barrier^28,29^. Furthermore, SG has shown activity in the CNS in clinical trials and real-world evidence studies^30–34^. In this post hoc analysis of 61 patients with stable treated brain metastases from the ASCENT study, SG showed numerically better outcomes than TPC for median PFS (2.8 vs 1.6 months) and tumor responses (3% vs 0%) but showed similar median OS to TPC. Though this represents only an incremental improvement in efficacy outcomes, whether SG is active across the blood–brain barrier in patients with progressive brain metastases remains unknown and is currently being investigated^35^.

These post hoc analyses in patients with age ≥65 years, Black race, or with brain metastases demonstrated that the safety profile of SG was consistent with the known AEs associated with SG in the ITT population^22^. The most commonly reported TEAEs across all patient subgroups were neutropenia and diarrhea, which were manageable with supportive care and dose reductions. Additionally, no cases of interstitial lung disease were reported with SG in this trial, an AE of concern with other classes of ADC agents commonly used to treat this patient population^36^. Treatment discontinuation due to TEAEs was generally low and there were no treatment-related deaths across patient subgroups. The safety profiles for individual TPC agents (eribulin, vinorelbine, capecitabine, and gemcitabine) were consistent with that of TPC overall^22^.

Data interpretation in these subgroup analyses is limited by the small sample size, therefore it can be difficult to draw firm conclusions from specific patient populations, especially patients with brain metastases. As all subgroup analyses were post hoc, no adjustment was made for multiple testing in the current analysis.

In conclusion, in these subgroup analyses from ASCENT, SG improved efficacy outcomes vs TPC in patient groups with mTNBC and poor prognosis in the second-line or later setting. In patients age ≥65 years, SG is safe and effective demonstrating improved clinical benefit over single-agent chemotherapy. In Black patients, a population historically known to have poor outcomes, SG offers an effective treatment option that improves survival outcomes over single-agent chemotherapy in this largest study of an ADC in patients with mTNBC to date. Efficacy outcomes across these subgroups and safety were consistent with that of the ITT population^22^. Although SG did not improve outcomes for patients with brain metastases compared with the ITT population, numerical PFS clinical benefit was observed in this extremely high-risk and difficult-to-treat population. These data also indicate that for patients with poorer survival outcomes, SG appears to have a greater benefit than TPC. Additionally, SG showed superior efficacy compared with each individual TPC chemotherapy. Taken together, SG should be considered an effective treatment option for patients with mTNBC eligible for second-line or later therapy.

## Methods

### Patients

Eligibility for the phase 3 ASCENT study (NCT02574455) has been reported previously^22^. Briefly, eligible patients had mTNBC (according to the standard American Society of Clinical Oncology/College of American Pathologists criteria; HER2 immunohistochemistry 0, 1, or 2/in situ hybridization negative; estrogen receptor/progesterone receptor <1%^37^) that was relapsed/refractory to ≥2 prior standard chemotherapy regimens (≥1 in the metastatic setting; no upper limit) for unresectable, locally advanced/metastatic disease. Patients were required to have received a prior taxane (any setting). Patients with known brain metastases (capped at 15%) were eligible if their central nervous system disease was stable for ≥4 weeks by MRI defined as ≥2 weeks from discontinuation of antiseizure medication and corticosteroid dose (≤20 mg prednisone equivalent) that was stable or decreasing for ≥2 weeks before randomization. Brain MRIs were required at each restaging throughout the study for these patients.

### Trial design and treatment

Details of the study design have been previously described^22^. Briefly, patients were randomized 1:1 to either SG (10 mg/kg intravenously on days 1 and 8, every 21 days) or TPC (capecitabine, eribulin, vinorelbine, or gemcitabine) until disease progression, unacceptable toxicity, study withdrawal, or death, whichever occurred first. No crossover to the SG arm was allowed upon progression with chemotherapy. Stratifications factors at randomization included number of prior chemotherapy regimens for advanced disease (2–3 vs >3) and presence of known brain metastases at baseline (yes vs no).

### Trial oversight

The study was approved by national regulatory authorities and each investigational site’s institutional review/ethics committee (see Supplement for full list of institutions) before implementation and was compliant with the Declaration of Helsinki and International Council for Harmonisation Good Clinical Practice Guidelines. As described in the originally reported study^22^, all patients provided written informed consent.

### Endpoints

The primary endpoint was PFS per BICR (using Response Evaluation Criteria in Solid Tumors [RECIST] v1.1^38^) in patients without known brain metastases. Secondary endpoints included PFS per investigator assessment, PFS in the ITT population (patients with and without brain metastases) by BICR, ORR, OS, and safety.

### Assessments

Tumor responses were assessed by imaging scans (computed tomography or MRI) obtained every 6 weeks for 36 weeks, then every 9 weeks thereafter, until disease progression requiring treatment discontinuation. Scans to confirm responses were required 4–6 weeks after initial assessment. During long-term follow-up, survival data were collected every 4 weeks.

Safety and tolerability were evaluated in all treated patients, with the severity of AEs graded using the National Cancer Institute Common Terminology Criteria for Adverse Events, version 4.03, and coded per the Medical Dictionary for Regulatory Activities, version 22.1. Premedication/concomitant medications and supportive measures allowed and recommended for patients during the study were previously described^22^.

### Subgroup analyses

Post hoc analyses described herein include subgroups defined by baseline demographics of age (age <65 years and ≥65 years), Black race, and brain metastases from the ITT population. The analysis of patients with no known brain metastases at baseline was prespecified in the study protocol. Analysis of outcomes with SG or TPC treatment were prespecified. Analysis by TPC selected prior to randomization was not prespecified per the protocol.

### Statistical analysis

Subgroup analyses were conducted using the statistical approach similar to the primary analysis^22^. Kaplan–Meier estimates were used to analyze PFS and OS in each treatment group, with medians and corresponding 95% CIs determined according to the Brookmeyer and Crowley method with log-log transformation (one-sided). The magnitude of the PFS and OS benefit was measured by HRs and their 95% CIs estimated from unstratified Cox proportional-hazards models. Response rate in each treatment group was reported together with the corresponding 95% CI based on the exact method.

Efficacy analyses were conducted in the patient population specified for each subgroup analysis. Efficacy in older and Black patients has previously been reported^22^. Safety was analyzed in patients who received one or more dose of study drug.

### Reporting summary

Further information on research design is available in the Nature Research Reporting Summary linked to this article.

### Supplementary information

Hurvitz ASCENT subgroups SG Supplement

Reporting Summary

## Data Availability

Gilead Sciences shares anonymized individual patient data upon request or as required by law or regulation with qualified external researchers based on submitted curriculum vitae and reflecting non conflict of interest. The request proposal must also include a statistician. Approval of such requests is at Gilead Science’s discretion and is dependent on the nature of the request, the merit of the research proposed, the availability of the data, and the intended use of the data. Data requests should be sent to datarequest@gilead.com.
