# Author Correction: Subgroup analyses from the phase 3 ASCENT study of sacituzumab govitecan in metastatic triple-negative breast cancer

**DOI:** 10.1038/s41523-024-00666-y

**Published:** 2024-07-09

**Authors:** Sara A. Hurvitz, Aditya Bardia, Kevin Punie, Kevin Kalinsky, Lisa A. Carey, Hope S. Rugo, Véronique Diéras, See Phan, Rosemary Delaney, Yanni Zhu, Sara M. Tolaney

**Affiliations:** 1 Clinical Research Division, Department of Medicine, UW Medicine, Fred Hutchinson Cancer Center, Seattle, WA USA; 2 Massachusetts General Hospital Cancer Center, Harvard Medical School, Boston, MA USA; 3 Department of General Medical Oncology and Multidisciplinary Breast Centre, Leuven Cancer Institute and University Hospitals Leuven, Leuven, Belgium; 4 Winship Cancer Institute, Emory University, Atlanta, GA USA; 5 University of North Carolina Lineberger Comprehensive Cancer Center, Chapel Hill, NC USA; 6 University of California San Francisco, Helen Diller Family Comprehensive Cancer Center, San Francisco, CA USA; 7 Centre Eugène Marquis, Rennes, France; 8 Gilead Sciences Inc., Foster City, CA USA; 9 Dana-Farber Cancer Institute, Harvard Medical School, Boston, MA USA

**Keywords:** Breast cancer, Breast cancer

Correction to: *npj Breast Cancer* 10.1038/s41523-024-00635-5, published online 25 April 2024

In Table 1 of this article, the ITT column had some incorrect values. These values have now been replaced with the correct values.

