# Supplementary material for: Subgroup analyses from the phase 3 ASCENT study of sacituzumab govitecan in metastatic triple-negative breast cancer
Source: NPJ Breast Cancer. 2024 Apr 25;10:33. doi: 10.1038/s41523-024-00635-5 (PMC11045722; doi:10.1038/s41523-024-00635-5)
Supplement: Supplementary file 1 — Hurvitz ASCENT subgroups SG Supplement [file 41523_2024_635_MOESM1_ESM.pdf]

SUPPLEMENTARY APPENDIX

This appendix has been provided by the authors to give readers additional information about their work.

Supplement to: Hurvitz et al. Subgroup analyses from the phase 3 ASCENT study of sacituzumab govitecan in metastatic triple-negative breast cancer

TABLE OF CONTENTS

Page Numbers

Supplementary Methods…List of institutions which provided ethical approval for ASCENT ………...**2**

Supplementary Table 1. Patient disposition by patient subgroup……………………………………….……..**4**

Supplementary Table 2. Summary of treatment-emergent adverse events (Safety Population; any grade [≥20%] or by worst grades ≥3 [≥5%] in the SG arm) – age subgroups………………………………..…..**6**

Supplementary Table 3. Summary of treatment-emergent adverse events (Safety Population; any grade [≥20%] or by worst grades ≥3 [≥5%] in the SG arm) – Black race subgroup……………………..…..**8**

Supplementary Table 4. Summary of treatment-emergent adverse events (Safety Population; any grade [≥20%] or by worst grades ≥3 [≥5%] in the SG arm) – brain metastases subgroup….…………**10**

Supplementary Table 5. Patient disposition by patient treatment group……………………..………….…**13**

Supplementary Table 6. Summary of treatment-emergent adverse events (Safety Population; any grade [≥20% of patients] or by worst grades ≥3 [≥5% of patients] in the SG arm) –by patient treatment subgroups…………………………………………………………………………………………………..………**14**

SUPPLEMENTARY METHODS

List of institutions which provided ethical approval for ASCENT:

Allegheny-Singer Research Institute, Pittsburgh, PA, USA; Barts Cancer Institute, Queen Mary University of London, London, United Kingdom; Beth Israel Deaconess Medical Center , Boston, MA, USA; Blue Ridge Cancer Care, Salem, VA, USA; Centre Eugène Marquis, Rennes, France; Centre Léon Bérard, Lyon, France; CHU Besançon, Hôpital Jean Minjoz, Besançon, France; CHU UCL Namur, Site Sainte Elisabeth, Namur, Belgium; Columbia University Irving Medical Center, New York, NY, USA; Complexo Hospitalario Universitario de Santiago (CHUS) – Hospital Clínico Universitario, A Coruña, Spain; Cross Cancer Institute, Edmonton, Alberta, Canada; Dana-Farber Cancer Institute, Boston, MA, USA; Facharztzentrum Eppendorf, Hamburg, Germany; Florida Cancer Specialists & Research Institute, Daytona Beach, FL, USA; Florida Cancer Specialists South, Fort Myers, FL, USA; Florida Cancer Specialists, Tampa, FL, USA; Georgetown Lombardi Comprehensive Cancer Center, Washington, DC, USA; Gianni Bonadonna Foundation, Milano, Italy; Hämatologisch-Onkologische Gemeinschaftspraxis am BethanienKrankenhaus, Frankfurt, Germany; Hospital del Mar, Barcelona, Spain; Hospital Teresa Herrera, A Coruña, Spain; Hospital Universitario 12 de Octubre, Madrid, Spain; Hospital Universitario Ramón y Cajal, Madrid, Spain; Hospital Universitario Virgen del Rocío, Sevilla, Spain; Illinois Cancer Specialists, Niles, IL, USA; Institut Catala d'Oncologia Hospitalet, Barcelona, Spain; Institut Claudius Regaud, IUCT – Oncopole, Toulouse, France; Institut Curie – Saint-Cloud, Saint-Cloud, France; Institut Curie, Paris, France; Institut de Cancérologie de l'Ouest (Site René Gauducheau), Saint-Herblain, France; Institut de Cancérologie de l'Ouest (Site René Gauducheau), Villejuif, France; Institut Gustave Roussy, Villejuif, France; Institut Jules Bordet, Université libre de Bruxelles, Brussels, Belgium; Institut Régional du Cancer de Montpellier, Montpellier, France; Instituto Oncológico Baselga – Hospital Quirónsalud Barcelona, Barcelona, Spain; International Breast Cancer Center (IBCC), Quiron Group, Barcelona, Spain; Leuven Cancer Institute, University Hospitals Leuven, Leuven, Belgium; Magee-Women's Hospital and the Hillman Cancer Center, University of Pittsburgh Medical Center, Pittsburgh, PA, USA; Maryland Oncology Hematology – Clinton Office, Clinton, MD, USA; Massachusetts General Hospital Cancer Center, Harvard Medical

School, Boston, MA, USA; Mayo Clinic, Rochester, MN, USA; Memorial Sloan Kettering Cancer Center, New York, NY, USA; Methodist Hospital, Houston, TX, USA; Miami Cancer Institute and Baptist Health South Florida, Miami, FL, USA; New York Oncology Hematology, PC, Albany, NY, USA; North Shore Hematology Oncology Associates, PC, Patchogue, NY, USA; Northside Hospital, Atlanta, GA, USA; Norwalk Hospital, Norwalk, CT, USA; Orlando Regional Medical Center, Orlando, FL, USA; Praxisklinik für Hämatologie und Onkologie Koblenz, Koblenz, Germany; Providence Cancer Center, Portland, OR, USA; Queen Elizabeth II Health Sciences Centre, Halifax, Nova Scotia, Canada; Rocky Mountain Cancer Centers, Greenwood Village, CO, USA; Rutgers Cancer Institute of New Jersey, New Brunswick, NJ, USA; Sarah Cannon Research Institute – Research Medical Center Kansas City, Kansas City, MO, USA; Segal Cancer Centre, Jewish General Hospital, Montreal, Quebec, Canada; Southern Cancer Center, Daphne, AL, USA; Surrey and Sussex Healthcare NHS Trust, East Surrey Hospital, Surrey, United Kingdom; Swedish Cancer Institute, Chicago, IL, USA; Sylvester Comprehensive Cancer Center, Plantation, FL, USA; Taunton and Somerset NHS Foundation Trust – Musgrove Park Hospital, Taunton, United Kingdom; Tennessee Oncology – Chattanooga, Chattanooga, TN, USA; Tennessee Oncology, Nashville, TN, USA; Texas Oncology – Baylor Charles A. Sammons Cancer Center, Dallas, TX, USA; Texas Oncology – Denton, Denton, TX, USA; Texas Oncology – Plano East, Plano, TX, USA; The Center for Cancer and Blood Disorders, Fort Worth, TX, USA; The Ohio State University Wexner Medical Center, Columbus, OH, USA; The Royal Free London NHS Foundation Trust – The Royal Free Hospital, London, 3 United Kingdom; The University of Chicago Medical Center, Chicago, IL, USA; Universitair Ziekenhuis Brussel, Brussels, Belgium; University Cancer & Blood Center, Athens, GA, USA; University Hospitals Coventry and Warwickshire NHS Trust, Coventry, United Kingdom; University of California San Francisco Helen Diller Family Comprehensive Cancer Center, San Francisco, CA, USA; University of California, Los Angeles, Jonsson Comprehensive Cancer Center, Los Angeles, CA, USA; University of Colorado Hospital – Anschutz Cancer Pavilion, Aurora, CO, USA; University of Kansas Cancer Center, Westwood, KS, USA; University of North Carolina Lineberger Comprehensive Cancer Center, Chapel Hill, NC, USA; US Oncology Research Pharmacy, Texas Oncology – Tyler, Tyler, TX, USA; Vall d'Hebron University Hospital and Vall d'Hebron Institute of Oncology (VHIO), Barcelona, Spain;

Vanderbilt-Ingram Cancer Center, Nashville, TN, USA; Virginia Cancer Specialists, PC, Fairfax, VA, USA; Virginia Oncology Associates, PC, Norfolk, VA, USA; Virginia Piper Cancer Institute Oncology Research, Minneapolis, MN, USA; Washington University School of Medicine in St. Louis, St Louis, MO, USA; West Cancer Center, Memphis, TN, USA.

SUPPLEMENTARY RESULTS

Safety Outcomes

Age

Patients age <65 years and those age ≥65 years treated with sacituzumab govitecan (SG) versus treatment of physician's choice (TPC) had similar rates of all grade and grade 3 or higher treatment-emergent adverse events (TEAEs).

The most common TEAEs of any grade for SG versus TPC included neutropenia (65% vs 44%), nausea (65% vs 31%), diarrhea (63% vs 16%), fatigue (51% vs 37%), and alopecia (49% vs 16%) for patients age <65 years and diarrhea (74% vs 19%), neutropenia (59% vs 44%), anemia (57% vs 31%), fatigue (53% vs 50%), and nausea (51% vs 29%) for patients age ≥65 years (Supplementary Table 2).

The most common grade ≥3 TEAEs for SG versus TPC included neutropenia (54% vs 32%), diarrhea (11% vs 1%), and leukopenia (11% vs 6%) for patients age <65 years and neutropenia (47% vs 40%), anemia (14% vs 6%), and diarrhea (12% vs 0%) for patients age ≥65 years.

TEAEs leading to dose reduction were similar in patients age ≥65 years in the SG versus TPC arms (37% vs 33%) and were lower in patients <65 years (19% vs 24%). Treatment discontinuation rates due to TEAEs with SG versus TPC were low in patients age ≥65 years (2% vs 2%) and < 65 years (5% vs 6%). There was one death in the age ≥65 years arm. Proactive AE monitoring and management will allow optimal therapeutic exposure to SG in older patients age ≥65 years.

Black Race and Other Race

For Black race, the most common TEAEs of any grade for SG versus TPC included diarrhea (72% vs 19%), neutropenia (64% vs 61%), nausea (52% vs 35%), and fatigue (52% vs 45%) (Supplemental Table 3).

The most common grade ≥3 TEAEs for SG versus TPC included neutropenia (48% vs 42%), anemia (12% vs 6%), leukopenia (8% vs 16%), and febrile neutropenia (8% vs 6%) (Supplemental Table 3). TEAEs leading to dose reduction were 28% in the SG arm and 36% in the TPC arm (Table 5). Treatment discontinuation rates due to TEAEs with SG versus TPC were low (4% vs 3%). There were no treatment-related AEs leading to death in the SG group.

For Other race, the most common TEAEs of any grade for SG versus TPC included diarrhea (64% vs 17%), neutropenia (64% vs 41%), nausea (64% vs 30%), and fatigue (52% vs 39%) (Supplemental Table 3).

The most common grade ≥3 TEAEs for SG versus TPC included neutropenia (53% vs 33%), anemia (9% vs 6%), leukopenia (11% vs 4%), and febrile neutropenia (6% vs 2%) (Supplemental Table 3). TEAEs leading to dose reduction were 22% in the SG arm and 25% in the TPC arm (Supplementary Table 1). Treatment discontinuation rates due to TEAEs with SG versus TPC were low (2% vs 3%).

Brain Metastases

The most common TEAEs of any grade for SG versus TPC included neutropenia (63% vs 52%), fatigue (63% vs 52%), diarrhea (50% vs 13%), and nausea (43% vs 26%; Supplementary Table 4). The most common grade ≥3 TEAEs for SG versus TPC included neutropenia (60% vs 26%), leukopenia (10% vs 9%), febrile neutropenia (10% vs 9%), and anemia (10% vs 4%). TEAEs leading to dose reduction were 17% in the SG arm and 35% in the TPC arm. TEAEs led to treatment discontinuation in 7% of patients in the SG arm and 9% in the TPC arm. There were no treatment-related AEs leading to death in the SG arm.

SG or TPC

The most common Grade ≥3 treatment-related adverse event with SG vs eribulin included neutropenia (52% vs 31%), leukopenia (11% vs 5%), diarrhea (12% vs 0%), anemia (9% vs 3%), febrile neutropenia (6% vs 3%), and fatigue (4% vs 7%) (Supplementary table 6). Vinorelbine, capecitabine, and gemcitabine information can be found in Supplemental Table 5.

Supplementary Table 1. Patient disposition by patient subgroup of age, race, or brain metastases[a]

	Age				Race				Brain Metastases			
	<65 years (*n* = 428)		≥65 years (*n* = 101)		Black (*n* = 62)		Other race (*n* = 467)		No (*n* = 468)		Yes (*n* = 61)	
	SG (*n* = 218)	TPC (*n* = 210)	SG (*n* = 49)	TPC (*n* = 52)	SG (*n* = 25)	TPC (*n* = 31)	SG (*n* = 233)	TPC (*n* = 193)	SG (*n* = 235)	TPC (*n* = 233)	SG (*n* = 32)	TPC (*n* = 29)
**Median duration of treatment exposure, mo (range)**	4.0 (0.0-29.6)	1.2 (0.0-15.3)	6.7 (0.3-24.6)	1.4 (0.1-8.1)	5.1 (0.0-29.6)	1.3 (0.0-15.3)	4.2 (0.03-29.6)	1.18 (0.03-10.6	5.1 (0.0-29.6)	1.3 (0.0-15.3)	2.5 (0.0-20.2)	1.2 (0.0-11.5)
**Treated, *n* (%)**	209 (96)	176 (84)	49 (100)	48 (92)	25 (100)	30 (97)	233 (100)	1887 (97)	228 (97)	195 (97)	30 (94)	23 (79)
**Remain on treatment, *n* (%)**	0	0	0	0	0	0	0	0	0	0	0	0
**Discontinued treatment, *n* (%)**	209 (96)	176 (84)	49 (100)	48 (92)	228 (97)	201 (86)	239 (100)	228 (100)	228 (97)	201 (86)	30 (94)	23 (79)
**Disease progression**	183 (84)	145 (69)	43 (88)	39 (75)	203 (86)	166 (71)	204 (85)	159 (70)	203 (86)	166 (71)	23 (72)	18 (62)
**Adverse event**	9 (4)	7 (3)	1 (2)	1 (2)	6 (3)	7 (3)	9 (4)	7 (3)	6 (3)	7 (3)	4 (13)	1 (3)
**Withdrawal of consent**	6 (3)	12 (6)	2 (4)	6 (12)	6 (3)	17 (7)	7 (3)	17 (8)	6 (3)	17 (7)	2 (6)	1 (3)
**Death**	0	4 (2)	1 (2)	0	1 (0.4)	4 (2)	1 (0.4)	4 (2)	1 (0.4)	4 (2)	0	0
**Treatment delay >3 weeks**	1 (0.5)	3 (1)	0	1 (2)	1 (0.4)	2 (1)	1 (0.4)	4 (2)	1 (0.4)	2 (1)	0	2 (7)
**Unacceptable toxicity**	0	0	0	1 (2)	0	1 (0.4)	0	1 (0.4)	0	1 (0.4)	0	0
**Physician decision**	9 (4)	5 (2)	2 (4)	0	10 (4)	4 (2)	10 (4)	1 (0.4)	10 (4)	4 (2)	1 (3)	1 (3)
**Other**	0	0	0	0	1 (0.4)	0	0	0	1 (0.4)	0	0	0

Other race subgroup includes any patient who did not self-identify as Black race. *SG* sacituzumab govitecan; *TPC* treatment of physician's choice.
[a]All patients who received one or more doses of study treatment (safety population).

Supplementary Table 2. Summary of treatment-emergent adverse events (Safety Population; any grade [≥20% of patients] or by worst grades ≥3 [≥5% of patients] in any treatment arm) – age subgroups.

		<65 Years				≥65 Years			
		SG (*n* = 209)		TPC (*n* = 176)		SG (*n* = 49)		TPC (*n* = 48)	
**TEAE[a], *n* (%)**		**All grade**	**Grade ≥3**	**All grade**	**Grade ≥3**	**All grade**	**Grade ≥3**	**All grade**	**Grade ≥3**
Hematologic	Neutropenia[b]	136 (65)	112 (54)	77 (44)	57 (32)	29 (59)	23 (47)	21 (44)	19 (40)
	Anemia[c]	75 (36)	17 (8)	47 (27)	10 (6)	28 (57)	7 (14)	15 (31)	3 (6)
	Leukopenia[d]	-	22 (11)	-	11 (6)	-	5 (10)	-	2 (4)
	Febrile neutropenia	-	11 (5)	-	6 (3)	-	4 (8)	-	0
Gastrointestinal	Diarrhea	132 (63)	24 (11)	29 (16)	2 (1)	36 (74)	6 (12)	9 (19)	0
	Nausea	136 (65)	--	54 (31)	--	25 (51)	--	14 (29)	--
	Vomiting	72 (34)	--	32 (18)	--	14 (29)	--	4 (8)	--
	Abdominal pain	43 (21)	--	15 (9)	--	12 (24)	--	3 (6)	--
	Constipation	78 (37)	--	43 (24)	--	18 (37)	--	9 (19)	--
Other	Fatigue	107 (51)	--	65 (37)	--	26 (53)	--	24 (50)	-
	Decreased appetite	57 (27)	--	37 (21)	-	14 (29)	--	9 (19)	--
	Asthenia	-	--	-	--	10 (20)	--	6 (13)	--
	Cough	50 (24)	--	31 (18)	--	11 (22)	--	9 (19)	--
	Headache	-	--	-	--	10 (20)	--	8 (17)	--
	Hypokalemia	-	--	-	--	10 (20)	--	11 (23)	--

	Dyspnea	-	--	39 (22)	-	-	4 (8)	-	2 (4)
	Alopecia	103 (49)	--	28 (16)	--	18 (37)	--	8 (17)	--

SG sacituzumab govitecan; *TEAE* treatment-emergent adverse events; *TPC* treatment of physician's choice.

[a]TEAE is defined as an adverse event with start date on or after the date of first dose of study treatment and up to 30 days after date of last dose of study treatment. Patients may report more than one per system organ class or preferred term. At each level of summarization, a patient is counted once if he/she reported one or more adverse events.

[b]Combined preferred terms of 'neutropenia' and 'neutrophil count decreased'.

[c]Combined preferred terms of 'anemia', 'hemoglobin decreased', and 'red blood cell count decreased'.

[d]Combined preferred terms of 'leukopenia' and 'white blood cell count decreased'.

- All grade adverse event not reported in ≥20% of patients in the SG arm or TPC arm

-- Grade ≥3 adverse event not reported in ≥5% of patients in the SG arm or TPC arm

Supplementary Table 3. Summary of treatment-emergent adverse events (Safety Population; any grade [≥20% of patients] or by worst grades ≥3 [≥5% of patients] in any treatment arm) – Race subgroups.

		Black				Other race			
		SG (*n* = 25)		TPC (*n* = 31)		SG (n = 258)		TPC (n = 224)	
**TEAE[a], *n* (%)**		**All grade**	**Grade ≥3**	**All grade**	**Grade ≥3**	**All grade**	**Grade ≥3**	**All grade**	**Grade ≥3**
Hematologic	Neutropenia[b]	16 (64)	12 (48)	19 (61)	13 (42)	149 (64)	123 (53)	79 (41)	63 (33)
	Anemia[c]	10 (40)	3 (12)	9 (29)	2 (6)	93 (40)	221 (9)	53 (28)	11 (6)
	Leukopenia[d]	6 (24)	2 (8)	10 (32)	5 (16)	--	25 (11)	--	8 (4)
	Febrile neutropenia	-	2 (8)	-	2 (6)	--	13 (6)	--	4 (2)
Gastrointestinal	Diarrhea	18 (72)	--	6 (19)	--	150 (64)	29 (12)	32 (17)	2 (1)
	Nausea	13 (52)	--	11 (35)	--	148 (64)	--	57 (30)	--
	Vomiting	9 (36)	--	9 (29)	--	77 (33)	--	27 (14)	--
	Abdominal pain upper	5 (20)	--	3 (10)	--	51 (22)	--	14 (7)	--
	Constipation	8 (32)	--	12 (39)	--	88 (38)	--	40 (21)	--
Other	Fatigue	13 (52)	--	14 (45)	-	120 (52)	11 (5)	75 (39)	16 (8)
	Hypophosphatemia	-	2 (8)	-	1 (3)	0	0	0	0
	Pulmonary embolism	-	2 (8)	-	0	0	0	0	0
	Decreased appetite	7 (28)	--	7 (23)	--	64 (28)	--	39 (20)	--

	Cough	7 (28)	--	5 (16)	--	54 (23)	--	35 (18)	--
	Alopecia	10 (40)	--	3 (10)	--	111 (48)	--	57 (30)	--

Other race subgroup includes any patient who did not self-identify as Black race. *SG* sacituzumab govitecan; *TEAE* treatment-emergent adverse events; *TPC* treatment of physician's choice.

[a]TEAE is defined as an adverse event with start date on or after the date of first dose of study treatment and up to 30 days after date of last dose of study treatment. Patients may report more than one per system organ class or preferred term. At each level of summarization, a patient is counted once if he/she reported one or more adverse events.

[b]Combined preferred terms of 'neutropenia' and 'neutrophil count decreased'.

[c]Combined preferred terms of 'anemia', 'hemoglobin decreased', and 'red blood cell count decreased'.

[d]Combined preferred terms of 'leukopenia' and 'white blood cell count decreased'.

- All grade adverse event not reported in ≥20% of patients in any treatment arm

-- Grade ≥3 adverse event not reported in ≥5% of patients in any treatment arm

Supplementary Table 4. Summary of treatment-emergent adverse events (Safety Population; any grade [≥20% of patients] or by worst grades ≥3 [≥5% of patients] in any treatment arm) – brain metastases subgroup.

		SG (*n* = 30)		TPC (*n* = 23)	
**TEAE,[a] *n* (%)**		**All grade**	**Grade ≥3**	**All grade**	**Grade ≥3**
Hematologic	Neutropenia[b]	19 (63)	18 (60)	12 (52)	6 (26)
	Anemia[c]	7 (23)	3 (10)	8 (35)	1 (4)
	Leukopenia[d]	-	3 (10)	-	2 (9)
	Thrombocytopenia	-	2 (7)	-	--
	Febrile neutropenia	-	3 (10)	-	2 (9)
Gastrointestinal	Diarrhea	15 (50)	2 (7)	3 (13)	0
	Nausea	13 (43)	--	6 (26)	--
	Vomiting	6 (20)	--	5 (22)	--
	Constipation	7 (23)	--	5 (22)	--
Other	Fatigue	19 (63)	--	12 (52)	-
	Back pain	6 (20)	--	7 (30)	-
	Sepsis	-	2 (7)	-	2 (9)
	Decreased appetite	9 (30)	--	4 (17)	--
	Hypokalemia	-	2 (7)	-	--
	Pneumonia	-	2 (7)	-	--
	Headache	7 (23)	--	3 (13)	--

	Mucosal inflammation	-	2 (7)	-	1 (4)
	Dyspnea	6 (20)	2 (7)	3 (13)	1 (4)
	Insomnia	6 (20)	--	2 (9)	--
	Alopecia	7 (23)	--	3 (13)	--

SG sacituzumab govitecan; *TEAE* treatment-emergent adverse events; *TPC* treatment of physician's choice.

[a]TEAE is defined as an adverse event with start date on or after the date of first dose of study treatment and up to 30 days after date of last dose of study treatment. Patients may report more than one per system organ class or preferred term. At each level of summarization, a patient is counted once if he/she reported one or more adverse events.

[b]Combined preferred terms of 'neutropenia' and 'neutrophil count decreased'.

[c]Combined preferred terms of 'anemia', 'hemoglobin decreased', and 'red blood cell count decreased'.

[d]Combined preferred terms of 'leukopenia' and 'white blood cell count decreased'.

[e]Combined preferred terms of 'leukopenia' and 'white blood cell count decreased'.

- All grade adverse event not reported in ≥20% of patients in any treatment arm.

-- Grade ≥3 adverse event not reported in ≥5% of patients in any treatment arm.

Supplementary Table 5. Patient disposition by patient treatment subgroup, SG or TPC

	Treatment				
		TPC			
	**SG (*n* = 267)**	**Eribulin (*n* = 139)**	**Vinorelbine (*n* = 52)**	**Capecitabine (*n* = 33)**	**Gemcitabine (*n* = 38)**
**Treated, *n* (%)**	258 (97)	123 (89)	41 (79)	28 (85)	32 (84)
**Remain on treatment, *n* (%)**	0	0	0	0	0
**Discontinued treatment, *n* (%)**	258 (97)	123 (89)	41 (79)	28 (85)	32 (84)
**Disease progression**	226 (85)	103 (74)	32 (62)	23 (70)	26 (69)
**Adverse event**	10 (4)	1 (1)	3 (6)	2 (6)	2 (5)
**Withdrawal of consent**	8 (3)	12 (9)	3 (6)	1 (1)	2 (5)
**Death**	1 (<1)	3 (2)	0	1 (3)	0
**Treatment delay > 3 weeks**	1 (<1)	2 (1)	0	0	2 (5)
**Unacceptable toxicity**	0	0	1 (2)	0	0
**Physician decision**	11 (4)	2 (1)	2 (4)	1 (3)	0

SG sacituzumab govitecan; *TPC* treatment of physician's choice.

Supplementary Table 6. Summary of treatment-emergent adverse events (Safety Population; any grade [≥20% of patients] or by worst grades ≥3 [≥5% of patients] in the SG arm) – by patient treatment subgroups

		**Treatment**					
		**SG (*n*=258)**		**TPC**			
				**Eribulin (*n* = 122)**		**Vin+Cap+Gem (*n* = 102)**	
**TEAE,[a] *n* (%)**		**All grade (n=257)**	**Grade ≥3 (n=188)**	**All grade (n=119)**	**Grade ≥3 (n=76)**	**All grade (n=100)**	**Grade ≥3 (n=69)**
Hematologic	Neutropenia[b]	165 (64)	135 (52)	48 (39)	38 (31)	50 (49)	38 (37)
	Anemia[c]	103 (40)	24 (9)	31 (25)	3 (3)	31 (30)	10 (11)
	Leukopenia[d]	NA	27 (11)	NA	6 (5)	NA	7 (7)
	Febrile neutropenia	NA	15 (6)	NA	3 (3)	NA	3 (3)
Gastrointestinal	Diarrhea	168 (65)	30 (12)	18 (15)	1 (1)	20 (20)	1 (1)
	Nausea	161 (62)	NA	43 (35)	NA	25 (25)	NA
	Vomiting	86 (33)	NA	22 (18)	NA	14 (14)	NA
	Abdominal pain	55 (21)	NA	7 (6)	NA	11 (11)	NA
	Constipation	96 (37)	NA	28 (23)	NA	24 (24)	NA
Other	Fatigue	133 (52)	11 (4)	48 (39)	8 (7)	41 (40)	11 (11)
	Decreased appetite	71 (28)	NA	23 (19)	NA	23 (23)	NA
	Cough	61 (24)	NA	23 (19)	NA	17 (17)	NA
	Dyspnea	45 (17)	10 (4)	27 (22)	7 (6)	20 (20)	5 (5)
	Alopecia	121 (47)	NA	32 (26)	NA	4 (4)	NA

Vin vinorelbine; *cap* capecitabine; *gem* gemcitabine.
