# Supplementary material for: Subgroup analyses from the phase 3 ASCENT study of sacituzumab govitecan in metastatic triple-negative breast cancer
Source: NPJ Breast Cancer. 2024 Apr 25;10:33. doi: 10.1038/s41523-024-00635-5 (PMC11045722; doi:10.1038/s41523-024-00635-5)
Supplement: Supplementary file 2 — Reporting Summary [file 41523_2024_635_MOESM2_ESM.pdf]

nature portfolio

Corresponding author(s): Sara Hurvitz

Last updated by author(s): Mar 25, 2024

Reporting Summary

Nature Portfolio wishes to improve the reproducibility of the work that we publish. This form provides structure for consistency and transparency in reporting. For further information on Nature Portfolio policies, see our Editorial Policies and the Editorial Policy Checklist.

Statistics

For all statistical analyses, confirm that the following items are present in the figure legend, table legend, main text, or Methods section.

n/a	Confirmed	
☐	☒	The exact sample size (*n*) for each experimental group/condition, given as a discrete number and unit of measurement
☒	☐	A statement on whether measurements were taken from distinct samples or whether the same sample was measured repeatedly
☐	☒	The statistical test(s) used AND whether they are one- or two-sided<br>*Only common tests should be described solely by name; describe more complex techniques in the Methods section.*
☒	☐	A description of all covariates tested
☐	☒	A description of any assumptions or corrections, such as tests of normality and adjustment for multiple comparisons
☐	☒	A full description of the statistical parameters including central tendency (e.g. means) or other basic estimates (e.g. regression coefficient) AND variation (e.g. standard deviation) or associated estimates of uncertainty (e.g. confidence intervals)
☒	☐	For null hypothesis testing, the test statistic (e.g. *F*, *t*, *r*) with confidence intervals, effect sizes, degrees of freedom and *P* value noted<br>*Give P values as exact values whenever suitable.*
☒	☐	For Bayesian analysis, information on the choice of priors and Markov chain Monte Carlo settings
☒	☐	For hierarchical and complex designs, identification of the appropriate level for tests and full reporting of outcomes
☒	☐	Estimates of effect sizes (e.g. Cohen's *d*, Pearson's *r*), indicating how they were calculated

Our web collection on statistics for biologists contains articles on many of the points above.

Software and code

Policy information about availability of computer code

Data collection	No software was used.
Data analysis	No software was used.

For manuscripts utilizing custom algorithms or software that are central to the research but not yet described in published literature, software must be made available to editors and reviewers. We strongly encourage code deposition in a community repository (e.g. GitHub). See the Nature Portfolio guidelines for submitting code & software for further information.

Data

Policy information about availability of data

All manuscripts must include a data availability statement. This statement should provide the following information, where applicable:

- Accession codes, unique identifiers, or web links for publicly available datasets
- A description of any restrictions on data availability
- For clinical datasets or third party data, please ensure that the statement adheres to our policy

The datasets used and/or analyzed during the current study are available from the corresponding author on reasonable request.

March 2021

Human research participants

Policy information about studies involving human research participants and Sex and Gender in Research.

Reporting on sex and gender	The majority of the patients in this study were women (99%+).
Population characteristics	Patients (n=529) diagnosed with mTNBC were enrolled. The median age was 54 years, and the median number of prior lines of systemic therapy was four. There were 101 patients (19%) age ≥ 65 years, 62 patients (12%) self-reported Black race, and 61 patients (12%) had known brain metastases at baseline.
Recruitment	Potential patients were screened from areas around trial site locations. Written informed consent was obtained by all participants.
Ethics oversight	FDA, EMA, Health Canada

Note that full information on the approval of the study protocol must also be provided in the manuscript.

Field-specific reporting

Please select the one below that is the best fit for your research. If you are not sure, read the appropriate sections before making your selection.

☒ Life sciences ☐ Behavioural & social sciences ☐ Ecological, evolutionary & environmental sciences

For a reference copy of the document with all sections, see nature.com/documents/nr-reporting-summary-flat.pdf

Life sciences study design

All studies must disclose on these points even when the disclosure is negative.

Sample size	Sample size for this post-hoc analysis was determined by selecting patients by age, self-reported race, and known brain metastases status at study entry. The sample sizes for these subgroups were small and thus it is difficult to state any firm conclusions.
Data exclusions	Data were not excluded.
Replication	This clinical trial was conducted in accordance with the approved study guidelines and as per the study protocol.
Randomization	Patients with mTNBC refractory were randomized 1:1 to receive SG or treatment of physician's choice (TPC) until unacceptable toxicity/ progression, withdrawal from the study, or death.
Blinding	The primary endpoint was PFS per blinded independent central review (BICR; Response Evaluation Criteria in Solid Tumors [RECIST] v1.1.

Reporting for specific materials, systems and methods

We require information from authors about some types of materials, experimental systems and methods used in many studies. Here, indicate whether each material, system or method listed is relevant to your study. If you are not sure if a list item applies to your research, read the appropriate section before selecting a response.

Materials & experimental systems

n/a	Involved in the study	
☒	☐	Antibodies
☒	☐	Eukaryotic cell lines
☒	☐	Palaeontology and archaeology
☒	☐	Animals and other organisms
☐	☒	Clinical data
☒	☐	Dual use research of concern

Methods

n/a	Involved in the study	
☒	☐	ChIP-seq
☒	☐	Flow cytometry
☒	☐	MRI-based neuroimaging

Clinical data

Policy information about clinical studies

All manuscripts should comply with the ICMJE guidelines for publication of clinical research and a completed CONSORT checklist must be included with all submissions.

Clinical trial registration	NCT02574455
Study protocol	A study protocol is available with this publication.
Data collection	Enrollment dates were from November 2017-September 2019 at 88 sites in seven countries.
Outcomes	We selected primary and secondary end points in line with other solid tumor cancer trials as is standard.
